# Chondrogenic commitment of human umbilical cord blood-derived mesenchymal stem cells in collagen matrices for cartilage engineering

**DOI:** 10.1038/srep32786

**Published:** 2016-09-08

**Authors:** Tangni Gómez-Leduc, Magalie Hervieu, Florence Legendre, Mouloud Bouyoucef, Nicolas Gruchy, Laurent Poulain, Claire de Vienne, Michel Herlicoviez, Magali Demoor, Philippe Galéra

**Affiliations:** 1 Normandy University, Caen, France; UNICAEN EA4652 MILPAT (Laboratoire Microenvironnement Cellulaire et Pathologies); UFR de Médecine, Université de Caen, Caen Cedex 5, CS14032 Caen, France; 2 Laboratoire de Cytogénétique Prénatale, Service de Génétique, CHU Caen, France; 3 Normandy University, Caen, France; UNICAEN, INSERM U1199 “Biology and Innovative Therapeutics of Locally Aggressive Cancers” Unit, Caen, France; 4 Service de Gynécologie-Obstétrique et Médecine de la Reproduction, CHU Caen, France

## Abstract

Umbilical cord blood (UCB) is a promising alternative source of mesenchymal stem cells (MSCs), because UCB-MSCs are abundant and harvesting them is a painless non-invasive procedure. Potential clinical applications of UCB-MSCs have been identified, but their ability for chondrogenic differentiation has not yet been fully evaluated. The aim of our work was to characterize and determine the chondrogenic differentiation potential of human UCB-MSCs (hUCB-MSCs) for cartilage tissue engineering using an approach combining 3D culture in type I/III collagen sponges and chondrogenic factors. Our results showed that UCB-MSCs have a high proliferative capacity. These cells differentiated easily into an osteoblast lineage but not into an adipocyte lineage. Furthermore, BMP-2 and TGF-β1 potentiated chondrogenic differentiation, as revealed by a strong increase in mature chondrocyte-specific mRNA (*COL2A1*, *COL2B*, *ACAN*) and protein (type II collagen) markers. Although growth factors increased the transcription of hypertrophic chondrocyte markers such as *COL10A1* and *MMP13*, the cells present in the neo-tissue maintained their phenotype and did not progress to terminal differentiation and mineralization of the extracellular matrix after subcutaneous implantation in *nude* mice. Our study demonstrates that our culture model has efficient chondrogenic differentiation, and that hUCB-MSCs can be a reliable source for cartilage tissue engineering.

Articular cartilage is a highly specialized avascular connective tissue composed of chondrocytes embedded within an intricate network of extracellular matrix (ECM). The latter is composed primarily of a type II collagen network and an interlocking mesh of fibrous proteins and proteoglycans, hyaluronic acid, and chondroitin sulfate. Due to its poor capacity for self-repair, cartilage is an ideal candidate for tissue engineering. Autologous chondrocyte implantation (ACI), described for the first time in 1994 by Brittberg *et al*., is the most widely used cell-based surgical procedure for the treatment of patients with focal cartilage damage^1^. This technique involves harvesting cartilage tissue from the patient, isolating chondrocytes from the donor tissue, expanding the cells *in vitro* and, finally, implanting the cells in the cartilage lesions. However, ACI has some limitations: it can cause significant donor-site morbidity, and chondrocyte expansion leads to cell dedifferentiation, potentially resulting in fibrocartilage formation after ACI treatment^2^. Mesenchymal stem cells (MSCs), once engaged in chondrogenesis, are a promising candidate for treating chondral defects. They are multipotent mesoderm-derived progenitor cells that were first identified in the bone marrow stroma (BM-MSCs)^3^ and have subsequently been isolated from a variety of other tissues, such as adipose tissue, umbilical cord and cord blood, and dental pulp^4^,^5^,^6^.

The International Society for Cellular Therapy (ISCT) stipulates the minimal criteria for defining MSCs: adherence to a plastic surface, expression of CD105, CD73 and CD90, but not CD45, CD34, CD14, CD19, and HLA-DR surface molecules, and ability to differentiate into osteoblasts, adipocytes or chondrocytes *in vitro*^7^. Due to their high potential for differentiation, MSCs are good candidates for tissue repair.

Many studies have focused on the potential of MSCs to differentiate into a chondrogenic lineage *in vitro*. Chondrocyte differentiation is based on three-dimensional (3D) high-density cell culture in pellets or on a variety of natural and synthetic scaffold materials in a defined chondrogenic medium containing conventional growth factors of the transforming growth factor-β (TGF-β) superfamily^8^,^9^. During *in vitro* chondrogenesis of MSCs, the transcription factor SOX9 promotes the transcription of genes encoding cartilage matrix proteins such as type II collagen and aggrecan^10^. Alternative splicing of type II collagen mRNA leads to two type II procollagen isoforms. The IIA isoform is expressed in early chondrogenesis, and the IIB isoform is expressed in mature articular cartilage^11^. Although *in vitro* chondrogenesis is possible, it is challenging because TGF-β also induces upregulation of hypertrophy-associated marker molecules such as type X collagen, matrix metalloproteinase-13 (MMP-13) and alkaline phosphatase, leading to matrix mineralization after ectopic transplantation in subcutaneous pouches in SCID-mice^12^. The *Col10a1* gene is expressed in pre-hypertrophic and hypertrophic chondrocytes in the growth plate and during osteoarthritis, but is not expressed by mature chondrocytes^13^. The challenge of cartilage engineering thus lies in producing complete and functional long-term MSCs differentiation without progression toward premature terminal hypertrophic differentiation and ossification.

Although, to date, adult bone marrow has been the main source of MSCs, harvesting them is a highly invasive procedure and the number of recovered progenitors, their differentiation potential^14^ and maximal life span decline with donor age^15^. These drawbacks clearly demonstrate the complexity and challenge of using MSCs as the candidate cell type for articular cartilage tissue engineering. Recently, an increasing number of studies have shown that specific MSC properties, including their chondrogenic differentiation capability, depend on their origin^16^,^17^. Umbilical cord blood-derived MSCs (UCB-MSCs) are a promising alternative source to bone marrow, because they are abundant, and harvesting them is a painless and non-invasive procedure. Despite limited studies on human UCB-MSCs (hUCB-MSCs), comparative analyses of proliferation and multilineage properties have shown that UCB-MSCs have several advantages over MSCs derived from bone marrow and adipose tissue^16^,^17^. Potential clinical applications of UCB-MSCs have been identified, but the potential for chondrogenic differentiation has not been yet fully evaluated nor have the effects of UCB-MSCs on cartilage repair^18^,^19^. Hence, it is important to determine the intrinsic differentiation capacity of UCB-MSC chondrocytes before using them for cell therapy or tissue engineering of cartilage.

A wide range of biomaterials have been evaluated for articular cartilage tissue engineering, they are generally based on either natural or synthetic polymers that can be processed into various forms including hydrogels, sponges, or fibrous meshes^8^. Although type II collagen is the major component of joint hyaline cartilage and would be an optimal scaffold for reconstruction of injured cartilage, the worldwide clinically practiced matrix-assisted autologous chondrocyte implantation procedure uses porcine-derived type I/III collagen bilayer membrane^20^. Type I and III collagens elicit a milder immune response than type II collagen since injection in rat used as model for rheumatoid arthritis induction^21^. Due to the weak antigenicity of type I collagen, it has been widely used from different animal origin as scaffold for many types of engineered tissues in humans^22^. Additionally, Ohnoa *et al*. showed that no significant differences in type I, type II and aggrecan gene expressions were found between seeded chondrocytes in either type I or II collagen sponges^23^. Furthermore, bovine articular chondrocytes cultured in type I/III collagen sponges do not express catabolic enzymes of cartilage, the metalloproteinases MMP-1 and MMP-13 in comparison to cells embedded in type I or type II collagen gels^24^. Claus *et al*. implanted type I/III collagen sponge–chondrocyte constructs cultured in human osteochondral blocks and then transplanted them subcutaneously in *nude* mice and observed that the constructs remained well integrated in the surrounding cartilage after the 6-weeks implantation period^25^. They have then demonstrated the feasibility of using cartilage re-constructed in collagen sponges for clinical application. In the present study, we used type I/III collagen from calf skin sponges for their chondrogenic potential and their compatibility with clinical practice^25^,^26^,^27^. This biodegradable collagen scaffold is mainly made with type I atelocollagen to remove the antigenic determinants and is chemically crosslinked to obtain a 3D scaffold with good mechanical and thermal stability^26^, which are essential characteristics for the use of biomaterials in tissue engineering therapy. In our previous work, this scaffold allowed attachment of rounded cells and chondrocyte-specific gene expression without any sign of hypertrophy or osteogenesis when human articular chondrocytes were seeded under low oxygen tension in the presence of BMP-2^28^. Thus, type I/III collagen sponges seem to be a suitable biomaterial for cartilage engineering and to investigate the behaviour of hUCB-MSCs differentiated into chondrocytes.

The aim of our study was to characterize hUCB-MSCs and to determine the chondrogenic differentiation potential of these cells for cartilage tissue engineering using an approach combining 3D-culture in type I/III collagen sponges and chondrogenic factors known to be efficient in chondrocyte redifferentiation. Ten markers of mature and hypertrophic chondrocytes and osteoblasts were studied to assess the functionality of the newly synthesized matrix *in vitro* and *in vivo* after implantation in *nude* mice. In addition, MSCs were characterized by evaluating several critical parameters: proliferation, karyotype, immunophenotype and differentiation into osteoblasts and adipocytes.

## Results

### Isolation and proliferation of hUCB-MSCs

**Figure 1:**
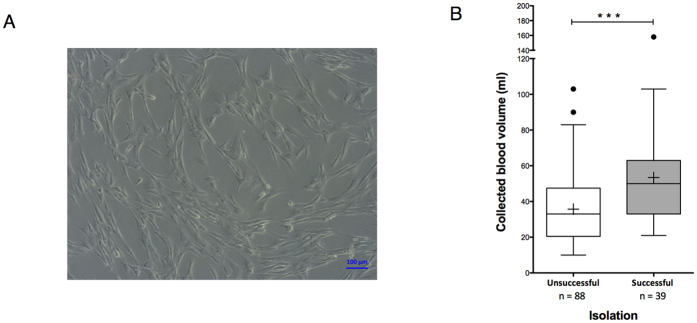
Morphology and isolation success of mesenchymal stem cells (MSCs) from human umbilical cord blood (hUCB). (**A**) Phase-contrast microscopy of adherent UCB-MSCs in culture (magnification ×10). (**B**) Correlation between collected volume and successful (fibroblastic cells able to form colonies) or unsuccessful (cells not able form colonies) isolation of UCB-MSCs. Data are presented as box plots. Individual outlying data points are shown as filled circles (**⚫**). Statistically significant differences between the unsuccessful and successful isolation of cells were determined using the Mann-Whitney U test. *Indicates statistically significant differences (***p < 0.001).

**Figure 2:**
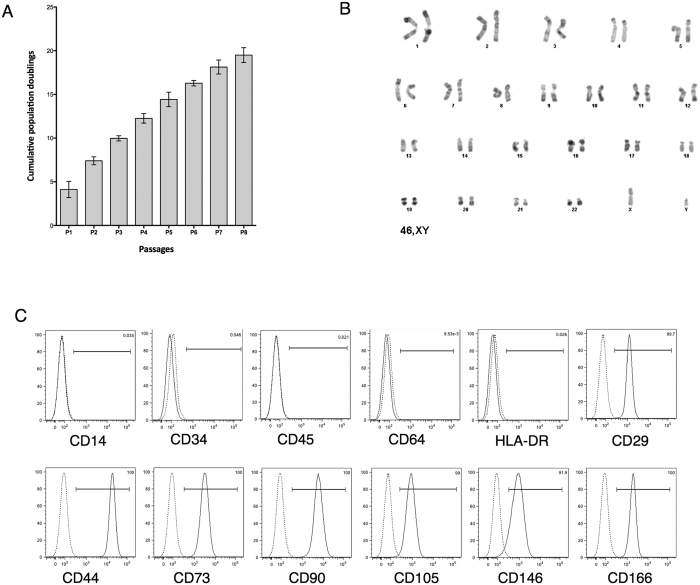
Analysis of proliferation capacity, karyotype and immunophenotype of human umbilical cord blood-derived mesenchymal stem cells (hUCB-MSCs). (**A**) Mean values of cumulative population doublings. Population doublings were determined at each passage of the adherent hUCB-MSCs. Graph represents mean ± standard deviation of eight hUCB donors. (**B**) Representative Q-banded karyotype analysis of hUCB-MSCs in culture at passage 5 shows genetic stability of the populations (n = 3). (**C**) Immunophenotype of hUCB-MSCs at passage 3. Each histogram is a representative result of at least three UCB-MSC samples. Dotted lines show the IgG isotype control response and solid line curves are for the samples.

We obtained 127 UCB samples with a volume ranging from 10 to 158 ml. Fibroblastic adherent cells formed in 39 UCB samples (Fig. 1A), representing an isolation success rate of approximately 30% (Fig. 1B). Taking into account only samples with a minimal volume of 55 ml, the success rate improved to 57%. The results suggest that the isolation success of MSCs depends on UCB volume. Adherent cells from eight hUCB-MSCs were passaged up to 8 times, and population-doubling (PDs) and cumulative population-doublings (CPDs) were calculated. We observed an increase in CPD at each passage with an average of 19.5±0.85 at P8 (Fig. 2A). The mean PD rate of hUCB-MSCs was greater than 2.5 up to P5. However, the doubling rate decreased significantly as of P6 in which we observed a change in cell morphology. In this study, *in vitro* hUCB-MSC amplification up to the fifth passage did not induce alteration in the number of chromosomes or any spontaneous structural chromosomal abnormality (Fig. 2B).

### Immunophenotypic characterization of hUCB-MSCs

The characterization of cell-surface markers on isolated hUCB-MSCs was performed with flow cytometry from P2 to P8. The results showed that cells were negative for the expression of hematopoietic markers such as CD14, CD34, CD45, CD64 and HLA-DR, but positive for several MSC markers including CD29, CD44, CD73, CD105, and CD166 (Fig. 2C). There was no significant variation in the expression of these markers during cell amplification (Table 1). hUCB-MSCs were also positive for the expression of CD90 and CD146, but had some particular features. CD90 expression was donor-dependent: either it was strongly expressed in hUCB-isolated cells or only in part of the cell population. Nevertheless, the CD90 expression profile remained unchanged during cell amplification. The expression of CD146, a surface marker on perivascular cells and pericytes, seemed to be donor- and passage-dependent in this study. During the early passages, the cells strongly expressed CD146 with a percentage of 51.3 ± 26.6 at P5, then, expression gradually decreased during the following passages, to an average of 26.5 ± 33.5 at P8 (Table 1).

### *In vitro* osteogenic and adipogenic hUCB-MSC differentiation capacity

**Figure 3:**
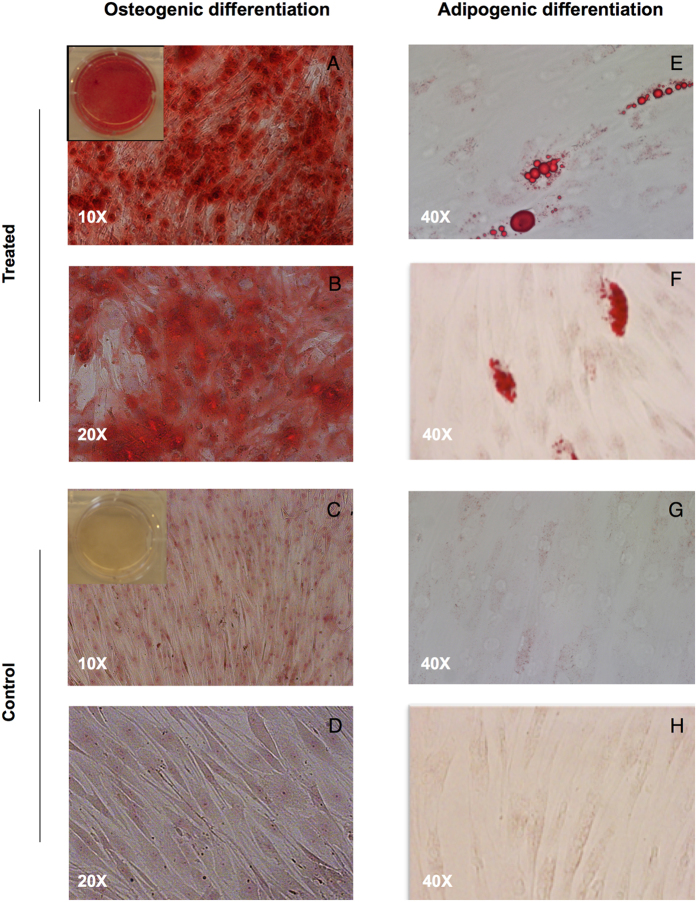
Osteogenic and adipogenic differentiation of human umbilical cord blood-derived mesenchymal stem cells (hUCB-MSCs). (**A**,**B**) After culture in osteoblastic induction medium, calcium mineralization was demonstrated by alizarin red S staining. A representative example from five adherent hUCB-MSC samples is shown (magnification ×10 and ×20). (**C**,**D**,**G**,**H**) Undifferentiated hUCB-MSCs. (**E**,**F**) After adipogenic induction and incubation in the maintenance medium, only a few cells show lipid droplets in the cytoplasm with oil red O staining. A representative example from three UCB donors is shown (magnification ×40).

To investigate hUCB-MSC differentiation potential *in vitro*, five hUCB-MSC samples were exposed to induction media for osteogenic or adipogenic differentiation for 3 weeks. After osteogenic induction, hUCB-MSCs showed ECM mineralization detected by alizarin red S staining in all samples (Fig. 3). Regarding adipogenic differentiation, only a few cells showed oil red O-stained droplets in their cytoplasm in three samples (Fig. 3). The two remaining samples did not show any stained lipid droplets, even after applying a 5 week fortified differentiation protocol, which comprised only the adipogenic induction medium in culture. No extracellular matrix mineralization or lipid droplets were detected in negative controls.

### Scanning electron microscopy of hUCB-MSCs seeded in type I/III collagen sponges

**Figure 4:**
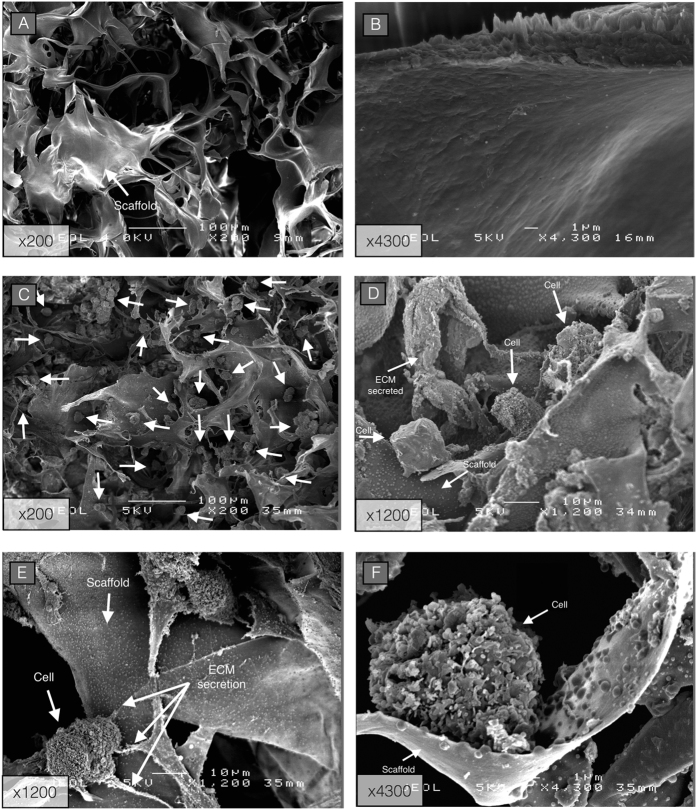
Scanning electron micrograph (SEM) images of both unseeded and seeded collagen sponge scaffolds. (**A**,**B**) Micrographs of a type I/III collagens sponge without cells (unseeded). (**C**–**F**) Micrographs taken in the internal area of a type I/III collagens sponge seeded with hUCB-MSCs during 7 days in incomplete chondrogenic medium. Magnification is shown on the lower left corner. Original magnification ×200 and scale bar: 100 μm. Original magnification ×1200 and scale bar: 10 μm. Original magnification ×4300 and scale bar: 1 μm.

SEM images of non-seeded collagen sponge scaffold showed a porous matrix with a pore size width of about 100 μm, allowing large spaces between collagen walls for cell growth (Fig. 4A). Collagen I/III sponges seeded with hUCB-MSCs and cultured for 7 days in ICM showed homogeneous cell distribution throughout the scaffold. hUCB-MSCs have a rounded shape in the superficial and internal areas of the scaffold (Fig. 4C–F).

### Expression of cartilage-specific and non-specific genes in hUCB-MSCs cultured in the collagen sponge scaffold

For chondrogenic differentiation, hUCB-MSCs were seeded in collagen sponge scaffolds and cultured in the presence or absence of BMP-2 and TGF-β1 for 14 days in normoxia. To evaluate the chondrogenic differentiation of hUCB-MSCs, RT-qPCR was performed for 10 genes. *SOX9*, *COL2A1*, *COL2A*, *COL2B* and *ACAN* encode characteristic proteins of native hyaline cartilage. *COL10A1*, *MMP-13* and *COL1A1* encode ECM molecules or enzymes of other cartilage types. *RUNX2* and *OSTEOCALCIN* encode bone markers. HAC mRNA extracts obtained from chondrocytes released from cartilage after an overnight enzymatic digestion were used as a control.

**Figure 5:**
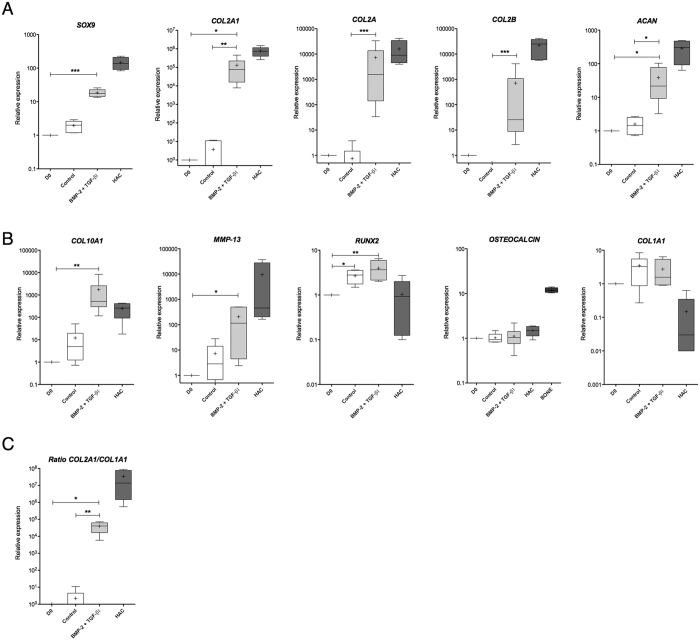
Effect of culture conditions on the mRNA steady-state levels of specific markers in mature and hypertrophic chondrocytes and osteoblasts. Human umbilical cord blood-derived mesenchymal stem cells (hUCB-MSCs) were cultured in collagen sponges at 21% O_2_ for 14 days, in incomplete chondrogenic medium in the absence (control) or presence of 50 ng/ml of BMP-2 and 10 ng/ml of TGF-β1. Undifferentiated hUCB-MSCs were also cultured as monolayers and used as a control before differentiation (day 0, D0). mRNA extracts obtained from human articular chondrocytes (HACs) released from cartilage after overnight enzymatic digestion were used as controls. (**A**) Relative mRNA expression of chondrogenic markers. (**B**) Relative mRNA expression of hypertrophic chondrocyte and osteoblast markers. Bone: mRNA extracted from osteoblast cultures at passage 3 and obtained from human femoral head of osteoarthritis patients. (**C**) *COL2A1*:*COL1A1* mRNA ratio. All results were normalized to *RPL13a* mRNA expression, compared with undifferentiated hUCB-MSCs cultured in monolayer, and presented as the relative expression of each gene. Box plots represent six independent experiments performed in triplicate. Statistically significant differences among hUCB-MSCs in monolayer and untreated or treated cells in sponges were determined using the Kruskal-Wallis test (*p < 0.05, **p < 0.01, ***p < 0.001).

In the absence of growth factors, hUCB-MSCs seeded in collagen sponge scaffolds exhibited only a slight increase in *SOX9*, *COL2A1*, *COL2A* and *ACAN* mRNA levels and no statistically significant differences were observed compared with undifferentiated cells (Fig. 5A). In contrast, treatment with growth factors upregulated expression of *SOX9* (9-fold), *COL2A1* (34,875-fold) and its isoforms *COL2A* (9695-fold) and *COL2B* (709-fold), and *ACAN* (24-fold) compared with the cells seeded in the collagen sponge scaffolds without growth factors. Interestingly, *COL2B*, a known marker of functionally mature chondrocytes was not expressed without growth factors (Fig. 5A). When, and only when, hUCB-MSCs were incubated with growth factors, the mRNA expression levels were very close to those observed in HACs. The committed hUCB-MSCs incubated in the presence of BMP-2 and TGF-β1 showed very strong potential for stage-specific chondrogenesis.

Subsequently, we tried to determine if culture conditions induce hUCB-MSC hypertrophy during chondrogenesis. *COL10A1* and *MMP-13* mRNA levels were significantly upregulated by 38-fold and 28-fold respectively in the presence of BMP-2 and TGF-β1 compared with the cells cultured in 3D in the absence of growth factors (Fig. 5B). These results suggest that growth factors enhance MSC hypertrophy during chondrogenic differentiation. Surprisingly, *MMP13* expression was lower in all groups compared to HACs. HACs, obtained from healthy femoral heads, had undergone enzymatic digestion of cartilage that may potentially affect *MMP13* expression. Type I collagen, considered as an unusual marker of hyaline cartilage, is constitutively expressed in undifferentiated hUCB-MSCs during monolayer amplification (data not shown). After 14 days in scaffold culture, *COL1A1* mRNA levels are upregulated by 3.5-fold and 2.7-fold in the absence and presence of growth factors respectively compared with undifferentiated hUCB-MSCs amplified in monolayer culture (Fig. 5B). No statistically significant differences were detected among the groups. The expression of *COL1A1* mRNA in monolayer and 3D culture remained higher compared with HAC levels. To better apprehend the chondrocyte phenotype, we also calculated the functional differentiation index corresponding to the ratio of *COL2A1* mRNA to *COL1A1* mRNA levels (Fig. 5C). The growth factors increased the *COL2A1*:*COL1A1* ratio by 17,999-fold compared with the control in collagen sponge scaffolds. Therefore, growth factors substantially improved the differentiated chondrocyte phenotype essential for the synthesis of a hyaline cartilage-like matrix.

Finally, the hUCB-MSC phenotypic profile after chondrogenic differentiation was further characterized by the analysis of *RUNX2* and *OSTEOCALCIN* mRNA steady-state levels (Fig. 5B). The culture in collagen sponges with or without growth factors slightly upregulated *RUNX2* compared with the undifferentiated hUCB-MSCs. *OSTEOCALCIN* mRNA was expressed in all culture conditions but at lower levels compared with osteoblasts used as a positive control. The increase in *RUNX2* expression suggests that cell hypertrophy occurred, but not osteoblastic differentiation. Furthermore, in collagen sponge cultured in normoxia, BMP-2 and TGF-β1 did not induce osteogenesis.

At the transcriptional level, the addition of BMP-2 and TGF-β1 to this culture model appeared to be the best way to induce hUCB-MSC chondrogenesis. However, these conditions also induced the expression of hypertrophy markers.

### Protein expression analysis during hUCB-MSC chondrogenesis induced by BMP-2 and TGF-β1

**Figure 6:**
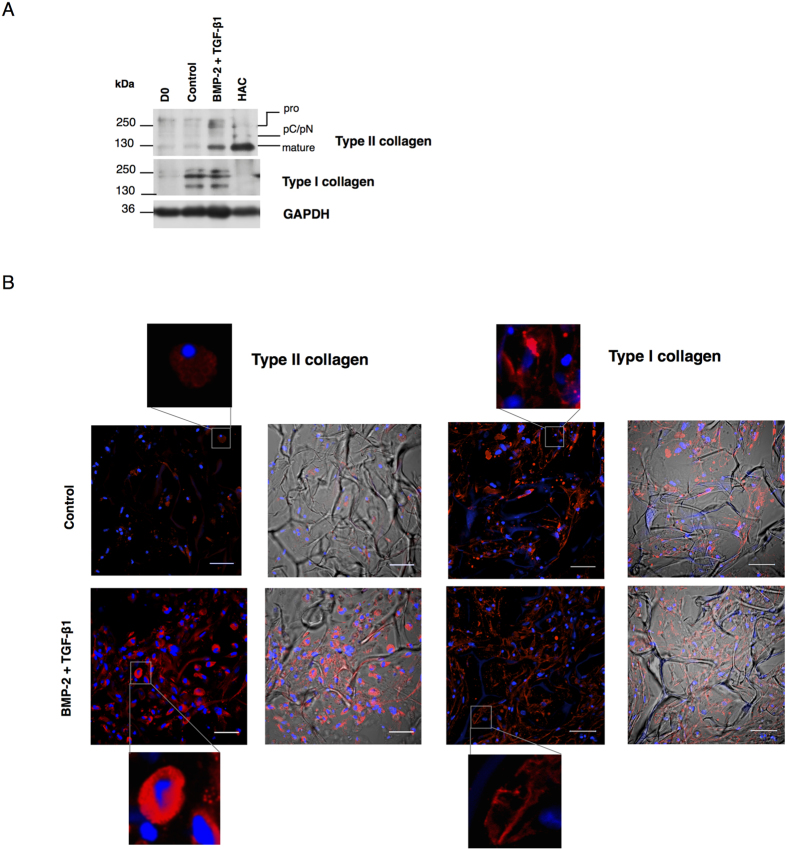
Effect of culture conditions on the protein expression of type I and II collagens. (**A**) Human umbilical cord blood-derived mesenchymal stem cells (hUCB-MSCs) were cultured in collagen sponges at 21% O_2_ for 14 days, in incomplete chondrogenic medium in absence (control) or presence of 50 ng/ml of BMP-2 and 10 ng/ml of TGF-β1. Undifferentiated UCB-MSCs were also cultured as monolayers and used as a control before differentiation (day 0, D0). (**A**) Protein extracts were analyzed in Western blots for type II and type I collagens *versus* GAPDH. Representative blots are shown (n = 6). Human articular cartilage (HAC) shows different levels of type II collagen maturation forms such as type II procollagen (pro), with only C- or N- terminal propeptides (pC/pN) and the doubly cleaved form (mature form). (**B**) Sponge constructs were obtained as described in panel A. hUCB-MSCs in sponges were fixed, permeabilized, and immunostained with anti-type II and anti-type I collagen rabbit antibodies. Goat anti-rabbit Alexa Fluor 546 secondary antibodies were used to visualize the localization of type I and II collagens (red). Nuclei were counterstained with DAPI (blue). Pictures were taken on a confocal laser-scanning microscope. Scale bar: 50 μm. For each collagen isotype left panels show fluorescence staining and the right panels, fluorescence staining merged with the corresponding light transmission image.

Protein extracts from human articular cartilage used in Western blots as a control showed different levels of type II collagen maturation forms such as type II procollagen (pro) with C and N-terminal propeptides, procollagen with only C or N-terminal propeptides (pC or pN) or the doubly cleaved form (mature form) (Fig. 6). After 14 days, we observed a different expression of type II collagen newly synthesized by hUCB-MSCs in the collagen sponge scaffolds in presence or absence of growth factors. Although bands corresponding to type II collagen were detected in hUCB-MSCs in monolayer culture or in collagen scaffolds without growth factors, they were less intense than in hUCB-MSCs treated with growth factors. When the cells were cultured with BMP-2 and TGF-β1, bands corresponding to pro, pN and mature forms of type II collagen were overexpressed. Type I collagen was expressed by cells seeded in collagen scaffolds with or without growth factors. Cartilage controls did not show any visible bands of type I collagen. Western blots (Fig. 6A) showed that BMP-2 and TGF-ß1 increased type II collagen synthesis. Type I collagen synthesis seems to be constitutive during sponge culture and appears very weakly decreased by growth factors treatment.

Immunofluorescence experiments were performed to visualize collagen synthesis in the sponge after 14 days of hUCB-MSC chondrogenic differentiation in culture. Images showed that in the presence of BMP-2 and TGF-β1, the type II collagen staining was more marked with a preferential localization in rounded cell cytoplasm. Type I collagen staining displayed a sparse network between cells, with a more marked fluorescence in the presence of BMP-2 and TGF-β1. In conclusion, protein analyses confirmed that chondrogenic differentiation is mainly induced in the presence of growth factors, whereas only modest type II collagen expression is observed in the absence of growth factors.

### *In vivo* neo-cartilage formation

**Figure 7:**
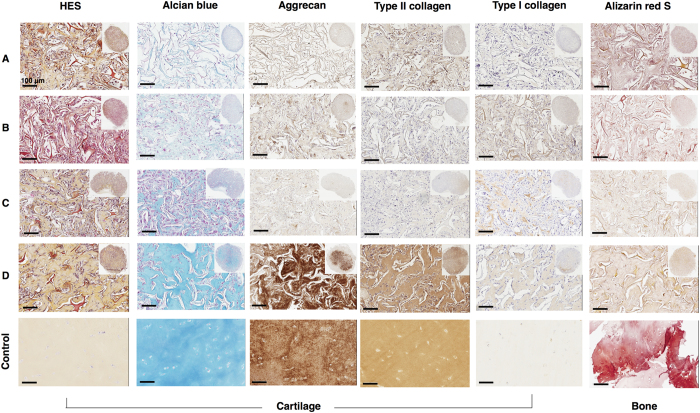
Histologic and immunohistochemical evaluation of ectopic cartilage-like transplants. Human umbilical cord blood-derived mesenchymal stem cells (hUCB-MSCs) were cultured in collagen sponges at 21% O_2_ for 14 days, in incomplete chondrogenic medium in the absence or presence of 50 ng/ml of BMP-2 and 10 ng/ml of TGF-β1. They were then implanted subcutaneously in *nude* mice and explanted 28 days later. Serial paraffin sections were stained with hematoxylin-eosin-safran, Alcian blue, analysed immunohistochemically for aggrecan, type II and I collagens, and alizarin red S-stained for calcium deposition. Parallel sections incubated without primary antibody were negative. (**A**) Empty sponge implanted in mice. (**B**) Undifferentiated UCB-MSCs cultured in collagen sponges for 24 hours before implantation. (**C**) UCB-MSCs cultured in collagen sponges in the absence of growth factors for 14 days before implantation. (**D**) UCB-MSCs cultured in the presence of BMP-2 and TGF-β1 for 14 days before implantation. (**Control**) Healthy human articular cartilage and human bone. The scale bar corresponds to 100 μm. The global aspect of the sponge construct, at lower magnification, is presented in the inset of the left images (**A**–**D**).

To evaluate the behavior of cells *in vivo*, after chondrogenic differentiation, sponge constructs of three different donors were implanted subcutaneously in the backs of *nude* mice for 4 weeks and then analyzed by histology and immunohistochemistry (Fig. 7). In parallel, we also grafted sponges that did not contain any cells. After 28 days, the sponge collagen network was still present. No degradation of the biomaterial occurred after 4 weeks of subcutaneous implantation in *nude* mice regardless of the culture conditions.

HES staining showed homogeneous cell distribution in sponges and an ECM, including sponges that initially contained no cells (empty sponges). This surprising finding suggests that murine cells invaded the empty collagen scaffold during the 28 days of incubation and synthesized their own ECM (Fig. 7A). Sponge constructs generated in the absence of growth factors were characterized by a large number of cells and a small amount of collagen. In contrast, when cells were treated with BMP-2 and TGF-β1, they showed lower cell numbers in the biomaterial and a homogeneous, abundant ECM.

We observed an enhanced staining for Alcian blue, an important marker of proteoglycan deposition, in cells treated with growth factors for 14 days before implantation when compared to undifferentiated hUCB-MSCs or in the absence of growth factors (Fig. 7).

Immunohistochemical analysis was performed to verify the cells had retained the chondrocyte phenotype observed *in vitro*, despite a discontinued supply of growth factors. Results showed that, in the absence of growth factors, type II collagen and aggrecan were slightly induced. In contrast, in the presence of BMP-2 and TGF-β1, the type II collagen expression was stronger and more homogeneous throughout most of the sponge. As shown for Alcian blue staining, aggrecan was also mainly localized to the central part of the sponge after treatment with growth factors.

Type I collagen staining displayed a sparse network between cells, with a more marked fluorescence in the presence of BMP-2 and TGF-β1. In addition, treatment with growth factors induced an ECM that appeared better organized and associated with a small number of cells, which matches the characteristics of hyaline cartilage tissue. In the presence of BMP-2 and TGF-β1, type I collagen levels were higher than control levels. However, type I collagen labelling was almost undetectable in some samples.

Finally, ectopic implantation of sponge constructs was also performed to verify if the extracellular matrix formed *in vitro* by the action of BMP-2 and TFG-β1 progresses to the matrix calcification. The ECM synthesized by hUCB-MSCs did not exhibit calcium deposition in the presence or absence of growth factors. These results suggest that the cells derived from UCB presenting a chondrocyte phenotype *in vitro*, after implantation for 28 days in *nude* mice, do not progress to an osteoblast phenotype characterized by ECM mineralization.

## Discussion

MSCs have been extensively studied for their promising potential in regenerative medicine. Nevertheless, MSCs show different characteristics according to their source and are not suitable for all regenerative medicine applications. In this study, we characterized and strove to assess whether UCB is a useful and accessible source of MSCs for cartilage engineering. To do so, we demonstrated the reliability of MSC isolation from human UCB and that these cells could be committed to the chondrogenic lineage using a culture approach combining 3D-culture in type I/III collagen sponges and chondrogenic factors.

The non-invasive nature of the samples and its wide availability make UCB a reliable MSC source. However, the success rate for isolating hUCB-MSCs is highly variable and controversies remain about the presence of MSCs in cord blood. Several studies have failed to isolate hUCB-MSCs^29^,^30^. We succeeded in isolating MSCs in 30% of all UCB samples, a rate similar to that reported in other studies^6^,^16^,^31^. However, our success rate was enhanced, reaching 57.1%, in sample volumes greater than 55 ml. Several studies have demonstrated that UCB volume is a crucial parameter that influences MSC isolation^6^,^32^. Zhang *et al*. obtained a success rate of 90% in hUCB-MSCs isolation with volumes of 90 ml and a maximum storage time of 2 h^32^. For some samples with high volumes, we were nevertheless unable to isolate MSCs, suggesting that other factors also influence the isolation success rate. For example, UCB of preterm deliveries seem to contain MSCs in sufficient concentrations^33^,^34^. One reason for the low number of studies on UCB compared to bone marrow is the time-consuming harvest process resulting in a low MSC isolation rate. However, applying strict criteria for UCB sample selection may result in higher efficiency, which can promote their use for therapeutic approaches.

For tissue engineering, an MSC-based approach requires a high cell number and thus sub-cultures. Despite an isolation time of one month (P0), the cells grow and multiply rapidly as of the first passage and we obtained a significant number of cells, even at early passages. Fetal cells, such as UCB-MSCs, provide cells with a homogeneous ontogenetic age, reduce variability and lead to more reproducible results in comparison with BM-MSCs, which are donor age-dependent^15^,^35^. However, we observed a decrease in proliferation during cell amplification and a marked reduction in the proliferation rate after the sixth passage. Previous reports indicate that MSCs have a limited life span and that after a certain number of cell divisions, the cells enter senescence, characterized by altered morphology and a decrease in proliferation as any normal somatic cell^36^. Likewise, our results suggest that cells must be used before the sixth passage to avoid the risk of cell senescence. Furthermore, long-term cultured MSCs can develop chromosomal abnormalities but without evidence of transformation potential^37^. These abnormalities can be avoided with methods that ensure slow growth and short expansion times because the number of expanded cells is linked to the growth rate, and a high proliferative rate may potentiate risks of karyotypic changes^38^. For this reason, we performed the differentiation experiments immediately after the third passage, when cells have a low cumulating-population levels and present a normal karyotype.

To study hUCB-MSC immunophenotype, we focused on ISCT recommended markers (CD73, CD90 and CD105)^7^ and other MSC markers well-described in the literature (CD29, CD44, CD146 and CD166)^6^,^17^,^31^,^39^. Our results showed that these membrane markers are expressed by cells in passage two and that their expression persists at least until the eighth passage. However, CD90 expression was donor-dependent, indicating that the hUCB-MSC population is heterogeneous, similar to BM-MSCs^40^. CD146 expression was also donor-dependent and decreased with cell passage. In agreement with our results, Sorrentino *et al*. showed that CD146^+^ population in hBM-MSCs was sharply reduced at P10 in association with a drop in the proliferation rate, but also with the differentiation potential and the hematopoietic support function^41^. Caplan’s team, in a recent review, refers to unpublished data suggesting that MSCs treatment by FGF-2, during *in vitro* amplification modifies the distribution of CD146^+^ cells. They propose that CD146 expression can be used to select cells with a maximum chondrogenic potential^42^. Further research will be necessary to elucidate whether there is a relation between expression of CD146 and the potential for differentiation into chondrocytes.

Eventhough MSCs have been defined as able to differentiate toward chondrocytes, osteoblasts and adipocytes^7^, the tripotence of UCB-MSCs was not demonstrated in this study. Although the cells differentiate easily and reproducibly into osteoblasts and chondrocytes, the differentiation into adipocytes was weak or absent. This observation corroborates previous reports showing that MSCs of fetal origin have a diminished adipogenic differentiation potential^16^,^43^.

Our results showed that the UCB cells have the capacity to differentiate into chondrocytes in collagen sponge scaffolds in the presence of growth factors. These results obtained *in vitro* are consistent with other studies that also show the strong ability of hUCB-MSCs to differentiate into chondrocytes. However, these studies used quite different protocols, hindering direct comparisons. Many of these studies were performed in *in vitro* monolayer cultures^31^,^43^, micromass or pellet cultures^6^,^16^,^44^ in the presence of a unique growth factor. Additionally, the chondrogenic differentiation was only revealed by staining proteoglycans or/and very few chondrocyte specific-markers. In a comprehensive study, the pellet culture of hUCB-MSCs in the presence of TGF-β1 (10 ng/ml) promotes RNA expression and secretion of types II and X collagen, SOX9, and aggrecan^39^. Although the pellet model allows cell-cell and cell-matrix contacts, which is important for chondrogenic differentiation, this 3D model limits oxygen and nutrient diffusion. The use of matrix scaffolds is a key component in the success of cartilage engineering. A number of recent studies have shown that UCB-MSCs are able to differentiate into chondrocytes in scaffolds^45^,^46^,^47^. For example, equine UCB-MSCs in membrane culture at low oxygen tension in the presence of TGF-β3 (10 ng/ml) produce a comparatively larger amount of high-quality neo-cartilage in comparison with differentiated cells in the pellet model^46^. However, the quality of the neo-synthesized matrix or mRNA expression in hUCB-MSCs were not compared with native tissue as in this study. Nonetheless, the nature of the scaffold can differently and directly influence cell behavior in culture^24^. Thus, further research with suitable scaffolds is required before their introduction in clinical practice. The collagen sponge scaffold used here for chondrogenic differentiation appears to be a good candidate for cartilage engineering. This collagen sponge scaffold, in contrast to pellets, is porous, allows the migration of seeded cells, and provides for more efficient nutrient, gas and waste diffusion. In addition, our collagen sponge scaffold allows a homogenous distribution of ECM and an abundant type II collagen content.

The impact of 3D collagen sponge culture of hUCB-MSCs in the absence or presence of BMP-2 (50 ng/ml) and TGF-β1 (10 ng/ml) was evaluated on the expression of several markers, considered as specific and non-characteristic for cartilage engineering applications. Cells seeded in the scaffold in the basal medium showed low expression of markers of the chondrocyte phenotype such as *SOX9*, *ACAN*, *COL2A1* and *COL2A* compared to cells cultured in monolayers. This spontaneous differentiation in the absence of growth factors can be explained by a high number of cells in the scaffold which allows cell-cell contact and mimics cell condensation during embryonic cartilage development^48^. In addition, the basal medium contains molecules commonly used to improve the synthesis of the ECM during chondrogenesis, such as dexamethasone, which may enhance the low induction of markers of the chondrocyte phenotype^49^. In summary, hUCB-MSC culture in a scaffold is not sufficient to properly induce chondrogenic differentiation and growth factors are essential to achieve MSC chondrogenesis *in vitro*. In this sense, the expression of SOX9, COL2A1, type II procollagen, IIB isoform and ACAN chondrogenic markers significantly increased when hUCB-MSCs were treated with BMP-2 and TGF-β1. In addition, the protein levels of type II collagen detected using Western blots showed that TGF-β1 and BMP-2 promote the mature form of the collagen. This result suggests that cells differentiated in the presence of these growth factors synthesize the molecules characteristic of hyaline type ECM and all the enzymes required for the maturation and assembly of collagens, leading to macromolecular organization of the ECM.

Differentiation of MSCs into chondrocytes, chondrocyte proliferation, chondrocyte hypertrophy and chondrocyte apoptosis are fundamental steps in skeletal development by endochondral ossification. Chondrocyte hypertrophy via endochondral ossification is used for osteoblast engineering but is undesirable for cartilage engineering^12^,^50^. In healthy articular cartilage, chondrocytes resist to proliferation and terminal differentiation to chondrocyte hypertrophy. In contrast, in diseased cartilage, chondrocytes proliferate and progressively develop hypertrophy. To obtain more information on the specific pathways regulating this chondrogenic differentiation, we analyzed *COL10A1* and *MMP-13* mRNA levels. Note that the interpretation of the type X collagen expression is difficult, because it is already expressed before induction of chondrocyte differentiation^51^. However, RT-qPCR studies performed showed that cells treated with BMP-2 and TGF-β1 have an increased *COL10A1* and *MMP-13* expression. Our results are consistent with other studies that have found that chondrogenic induction in MSCs is related to an increase in hypertrophy-associated markers^12^,^39^,^52^. Despite the expression of hypertrophy markers in our study at the mRNA level, we have not observed a matrix mineralization after ectopic implantation in mice. Further investigations are necessary to find solutions to prevent hypertrophic maturation of cells *in vitro*. A recent study demonstrated that oxygen tension plays an important role in controlling the differentiation of MSCs. Low oxygen tension decreases the expression of the markers of terminal differentiation in BM-MSC cultures during chondrogenic differentiation in the presence of BMP-2 and/or TGF-β3^53^,^54^. Similarly, thrombospondin-2 attenuates hypertrophy, which occurs during chondrogenic differentiation of hUCB-MSCs^55^. Another study indicates that the addition of FGF-9 and FGF-18 at day 14 of chondrogenic differentiation delays the appearance of the hypertrophic phenotype^56^. Taken together, these strategies can be used to optimize pre-implantation conditions of MSCs for engineered cartilage grafts.

We evaluated if hUCB-MSCs are able to differentiate into chondrocytes or osteoblasts. TGF-β1, -β3 and, BMP-2 are the most extensively used growth factors for inducing MSC chondrogenesis *in vitro*^16^,^44^,^56^. However, BMP-2 is also used for the differentiation of stem cells into osteoblasts^57^. For this reason, we evaluated the expression of the transcription factors *SOX9* and *RUNX2*, associated with a commitment of MSCs to the chondrocyte and osteoblast phenotypes, respectively. We showed that our culture model allows MSC commitment to the chondrocyte phenotype with an increased expression of *SOX9* in the presence of growth factors, whereas the expression of *RUNX2* was low and may correspond to cell hypertrophy during chondrogenic differentiation. For example, Runx2 *trans*activates type X collagen and matrix metalloproteinase-13 gene transcription in hypertrophic chondrocytes^58^,^59^. The low expression of *OSTEOCALCIN* mRNA suggests that our culture model therefore promotes mainly the differentiation of cells into chondrocytes.

Our MSC-seeded constructs were implanted in *nude* mice subcutaneously for an additional 28 days after 14 days of chondrogenic induction *in vitro*. We observed a homogenous ECM with abundant type II collagen and aggrecan production. This observation indicates that constructs continued to mature during the implantation period in *nude* mice. This model represents a preliminary step for guaranteeing phenotypic stability and suitability of these cells for the repair of cartilage defects.

Our results are promising and demonstrate that cells maintain their phenotype and do not continue to terminal differentiation and ECM mineralization. Human BM-MSCs cultured in identical conditions are subject to a matrix calcification after subcutaneous transplantation in *nude* mice (unpublished data). Remarkably, we found that 2 weeks of pre-induction of hUCB-MSCs was sufficient to recover ectopic cartilage-like tissue at later stages. These results suggest stable ectopic cartilage formation and that hUCB-MSCs are a very promising source for the tissue engineering of articular cartilage. Collagen sponges also appear to be a suitable biomaterial for cartilage engineering. Nevertheless, we are aware that the mouse subcutaneous ectopic environment is not representative of the physiological situation in articular cartilage. In the natural joint environment, MSCs may behave differently, and other animal models must be investigated for pre-clinical trials. Furthermore, a hyaluronic acid hydrogel in a articular cartilage defect rat model has been shown to be a feasible solution for cartilage repair using hUCB-MSCs^18^,^19^. However, natural scaffolds in the form of hydrogels have poor biomechanical properties, limiting their application in tissue engineering. In addition, the success of human cell transplantation needs to be evaluated in appropriate cartilage-defect models in larger animals such as the horse, which is an excellent model of articular pathology. This model is particularly useful because cartilage injury is the most common cause of lost training days or premature retirement in the equine athlete.

Taken together, we set out to demonstrate that hUCB-MSCs can be an alternative choice for the treatment of cartilage defects when they are seeded in a collagen sponge scaffold in the presence of BMP-2 and TGF-β1. However, there are still some limitations to our approach and we need to take them into account for our future studies. We induced chondrogenic differentiation for only 14 days in the presence of BMP-2 and TGF-β1 in normoxia without establishing conditions for chondrocyte phenotype stabilization. We used BMP-2 and TGF-β1 together. However, the role of each factor individually during differentiation is still poorly understood and BMPs and TGF-β isoforms have been described to exhibit antagonistic activities^60^. The method described here may be useful to test extended culture times and oxygen conditions, and help explain the mechanisms and optimize the desired response.

**In conclusion**, we demonstrated that our culture model allows for efficient chondrogenic differentiation, and that hUCB-MSCs may be a reliable source for cartilage tissue engineering. In addition, this study contributes to the development of future strategies in effective tissue engineering using UCB-MSCs for the repair/regeneration of damaged cartilage tissue.

## Materials and Methods

### Cell isolation and cell culture

hUCB was collected from umbilical veins after neonatal delivery, immediately after full-term deliveries. We collected 127 hUCB samples after written informed consent from all the mothers according to the hospital’s human ethics committee guidelines, at the Obstetrics and Gynecology Unit (“*Femme-Enfant-Hématologie*” Department at the *Centre Hospitalier Universitaire* in Caen, France). All experiments were performed in accordance with relevant, approved guidelines and regulations. All experimental protocols were approved by the local Ethics Committee for research with human samples (Comité de Protection des Personnes Nord Ouest III) of the *Centre Hospitalier Universitaire* of Caen.

The hUCB samples were collected into sterile flasks containing 17 ml of citrate phosphate dextrose anticoagulant, stored at room temperature, and processed within 1 to 6 h after collection. To isolate mononuclear cells (MNCs), each UCB unit was diluted 1:1 with phosphate-buffered saline (PBS) and carefully mixed with Ficoll-Paque PREMIUM (GE Healthcare Bio-Sciences) medium. After density gradient centrifugation at 400 × g for 30 min at room temperature, MNCs were washed once with PBS. UCB-derived MNCs were seeded in culture flasks in low glucose-Dulbecco’s modified Eagle Medium (LG-DMEM; Invitrogen) containing 20% fetal calf serum (FCS, Invitrogen Life Technologies), 10^−7 ^M dexamethasone (Sigma-Aldrich) and incubated at 37 °C in a 5% CO_2_ atmosphere. A cocktail of antimicrobials composed of 100 IU/ml of penicillin, 100 μg/ml of erythromycin, and 0.25 mg/ml of fungizone was added to all the media used in this study. Non-adherent cells were removed 24 h after initial plating. The medium was changed twice weekly until adherent cells appeared, defined as passage zero (P0). After the appearance of several colonies in 39 samples, cells were detached using trypsin/EDTA (Invitrogen) and then reseeded at 5 000 cells/cm^2^ (passage one, P1) and thereafter. Cell expansion was performed in the same media without dexamethasone. The culture medium was changed twice a week and cells were passaged at 80% confluency until passage 8 (P8). The 8 samples used for further experiments were chosen at random between 39, but they were selected on the basis of minimal criteria of ISCT defining MSCs: adherence to a plastic surface, PDs-CPDs, expression of MSCs markers and absence of expression of hematopoietic CDs (Supplementary Table 1).

Human articular chondrocytes (HAC) were prepared from macroscopically healthy zones of femoral heads of patients undergoing joint arthroplasty at the Dept. of Surgery at St Martin Clinic (Caen, France)^26^,^27^. All patients signed an informed consent agreement form, which was approved by the local human research ethics committee (*Comité de Protection des Personnes Nord Ouest III*). Cartilage samples were cut into small slices, then chondrocytes were isolated by sequential digestion for 45 min at 37 °C with 2 mg/ml of type XIV protease (Sigma) and then overnight at 37 °C with 1 mg/ml of type I collagenase (from *Clostridium histolyticum*; Invitrogen) as previously described^26^,^27^. The cell suspension was filtered through a 70 μm mesh nylon membrane and centrifuged at 200 × g for 10 min. The pelleted cells were re-suspended in Trizol (Invitrogen) and RNA extraction was carried out according to the manufacturer’s protocol. For Western-blot experiments, small cartilage slices were ground in liquid nitrogen and protein extraction was performed with RIPA buffer. HAC extracts were used in real-time reverse transcription-polymerase chain reaction (RT-PCR) and Western-blot as controls.

The population-doubling level was calculated for each passage (from P1 to P8) according to the following equation: population doublings = [log10(NH) − log10(NI)]/log10(2), where NI is the number of inoculated cells and NH is the number of harvested cells. Then, the cumulative population-doubling increase was added to the PD levels of the previous passages to obtain the cumulative population-doubling level (CPD) = ΣPD.

### Karyotype analysis

Karyotyping was performed on cells at P5 by the Cytogenetics Department of the hospital. Briefly, hUCB-MSCs were cultured in medium supplemented with colcemid (10 μg/ml, PAA Laboratories). Then, the cells were re-suspended in a hypotonic solution of KCl and incubated at 37 °C for 10 min. After centrifugation, cells were resuspended in a fixative solution (3:1 methanol:acetic acid). Metaphase spreads were then prepared on glass microscope slides and analyzed using R- and Q-banding Giemsa staining. A total of 16 mitoses were analyzed per sample using CytoVision software (Leica Biosystems).

### Immunophenotyping

hUCB-MSCs (from P2 to P8) were harvested, washed, and resuspended in PBS-sodium azide buffer (0.1%) at a density of 10^6 ^cells/ml. Cell suspensions were incubated with monoclonal antibodies for 25 min at 4 °C in a dark room. The following mouse monoclonal anti-human antibodies were used: CD14-Fluorescein isothiocyanate (FITC), CD34-allophycocyanin (APC), CD45-peridinin chlorophyll protein complex (PerCP), CD64-phycoerythrin (PE), CD73-PE, CD146-PE, CD166-PE, HLA-DR-FITC (BD Pharmigen), CD29-FITC, CD44-PE, CD90-PE (IOTest) and CD105-FITC (ABd Serotec). The respective mouse isotype antibodies served as controls. Subsequently, the cells were washed and resuspended in 300 μl PBS-azide. Data acquisition was performed in a BD FACS Canto II flow cytometer (BD Biosciences) and analyzed with FlowJo Software (TreeStar).

### Osteoblastic and adipogenic differentiation

The capacity of hUCB-MSCs to differentiate in osteogenic and adipogenic lineages was determined at P4. For this purpose, cells were seeded in 6-well plates. All the reagents used in the osteogenic and adipogenic differentiation experiments were purchased from Sigma.

Osteogenic differentiation was initiated when cells reached 70% of confluence in LG-DMEM supplemented with 10% FCS, 10^−7 ^M dexamethasone, 100 μM ascorbic acid-2-phosphate, and 10 mM β-glycerophosphate. The culture medium was changed once a week and the cells were incubated for 3 weeks. Osteogenic differentiation was assessed by alizarin red S staining. Briefly, cells were fixed with 4% paraformaldehyde for 10 min at room temperature, washed once with PBS (pH 4.1) and stained for 20 min with alizarin red S at room temperature.

Adipogenic differentiation was induced when cells reached 90% confluency. Induction was obtained by performing three cycles of culture in induction (for 72 h) and maintenance (for 24 h) medium. The induction medium consisted of LG-DMEM supplemented with 10% FCS, 10^−6 ^M dexamethasone, 0.5 mM 3-isobutyl-1-methyl-xanthine, 0.2 mM indomethacin, and 10 μg/ml recombinant human (rh) insulin. The maintenance medium consisted of LG-DMEM supplemented with 10% FCS and 10 μg/ml rh insulin. After the three cycles of induction and maintenance, cells were incubated for another 7 days in adipogenic maintenance medium. Adipocytes were revealed by intracellular accumulation of lipid vacuoles using oil red O staining and identified by their bright red color. Briefly, cells were fixed, washed and stained with 0.3% oil red O in isopropanol and incubated for 10 min at room temperature. As negative controls for osteogenic and adipogenic differentiation, cells were cultured in LG-DMEM supplemented only with 10% FCS.

### Chondrogenic differentiation

The scaffold was manufactured by Symatèse Biomatériaux (Chaponost, France). These collagen sponges (100% of collagen, 2 mm thickness, 5 mm diameter, corresponding to a volume of 0.04 cm^3^, around 100 μm pore size) are composed of native type I (90–95%) and type III (5–10%) collagens from calf skin; they are crosslinked with glutaraldehyde to increase their stability. They are sterilized with β-radiation and they do not swell after rehydratation.

UCB-MSCs were grown for 14 days in a 3D scaffold to induce chondrogenesis according to the following protocol. Briefly, cells were subcultured as monolayers until P3, trypsinized, and suspended in incomplete chondrogenic medium (ICM) composed of high glucose-DMEM (4.5 g/L) supplemented with 50 μg/ml ascorbic acid-2-phosphate, 100 μg/ml sodium pyruvate, 40 μg/ml proline (Fluka), 1:100 dilution of insulin transferin selenium (Invitrogen) and 10^−7 ^M dexamethasone. Cell seeding onto the collagen sponges was performed by dropping 20 μl of the cell suspension on the sponge (5 × 10^5 ^cells/sponge) in 96-well culture plates and incubating the plates at 37 °C under 5% CO_2_. After 1 h, the cell constructs were transferred to 24-well plates with ICM in presence or absence of 50 ng/ml bone morphogenetic protein-2 (rhBMP-2, inductOs to Wyeth Europa Ltd) and 10 ng/ml rhTGF-β1 (Miltenyi Biotec). Constructs were incubated at 37 °C under 5% CO_2_ and the medium was changed on days 4, 7, 11. Sponges were harvested for RNA and protein analyses on day 14. hUCB-MSC monolayers before induction were used as controls (day zero, D0).

For *in vivo* studies, hUCB-MSCs from three different donors were seeded in collagen sponges, incubated in ICM with or without BMP-2 and TGF-β1 for 14 days as described above. The *nude* mice (Athymic nude-foxn1, female, 4 weeks, Harlan, France) were anesthetized and four sponges were implanted subcutaneously in the mouse dorsal area. Animal experiments were approved by the Regional Ethics Committee (CENOMEXA 1011-01) and were performed in accordance with institutional animal guidelines. Surgical procedures were performed under general anesthesia with inhalation of 4% isoflurane. After 28 days, *nude* mice were euthanized under anesthesia (5% isoflurane) with CO_2_ inhalation and the constructs were surgically removed. Then, histological and immunochemical analysis was performed.

### Scanning electron microscopy

hUCB-MSCs cultured in collagen sponges incubated in ICM for 7 days were fixed for 24 h with 2.5% glutaraldehyde in PBS at 4 °C, and washed by 0.1 M phosphate buffer, pH 7.4. Samples were postfixed with 1% osmic acid for 2 h, washed, and dehydrated in a graded series of ethanol. Then, they were critical point-dried, coated with a platinum layer for scanning electron microscopy (SEM) observations (JEOL 6400F). Non-seeded scaffolds were also cut and sputter-coated with platinum and analyzed by SEM. Non-seeded scaffolds were subjected to the same conditions as the seeded scaffolds (culture in ICM for 7 days) before SEM analysis.

### RNA isolation and RT-qPCR

After treatment, sponges seeded with cells were rinsed once with ice-cold phosphate-buffered saline and total RNA was extracted using Trizol Reagent according to manufacturer’s instructions. One microgram of RNA was reverse transcribed into cDNA using reverse transcriptase (MMLV, Invitrogen) and oligodT (Eurogentec). PCR was performed on an Applied Biosystems 7700 Real-Time system using the TaqMan PCR Master Mix (Applied Biosystems) for *COL2B* collagen and using Power SYBR Green PCR (Applied Biosystems) for the other genes, as previously described. Sequences of the primers and probe used are listed in Table 2. Relative gene expression was calculated using the 2^−ΔΔCT^ method and expressed as the mean of triplicate samples. Each sample was normalized to *RPL13a* and each group was normalized to the expression levels of undifferentiated hUCB-MSCs.

### Western blots

After treatment, sponges containing cells were rinsed once with ice-cold PBS, crushed and total proteins were extracted from cells using the RIPA-lysis buffer with a protease inhibitor cocktail. Protein concentration was assessed according to the Bradford colorimetric procedure (Bio-Rad SA). Then, 20 μg of total proteins were separated in 10% polyacrylamide gels containing 0.1% SDS and transferred to a polyvinylidene difluoride membrane (PVDF, Millipore). Unspecific binding sites of the membrane were blocked with 10% non-fat milk powder in Tris-buffered saline with 0.1% Tween (TBST) for 2 h. Then, membranes were incubated overnight at 4 °C with rabbit anti-human type I collagen (Novotec), rabbit anti-human type II collagen (Novotec) or rabbit anti-human GAPDH (Santa Cruz Biotechnology, Inc.). The following day, membranes were washed three times, followed by an incubation with HRP-conjugated goat anti-rabbit IgG antibody (Jackson Immunoresearch). Signals were visualized with the chemiluminescence method (ECL plus western blotting detection reagent+, Santa Cruz Biotechnology, Inc.) and developed on X-ray film.

### Immunofluorescence

After 14 days of chondrogenesis induction, sponge constructs were rinsed with 0.1 M phosphate buffer, pH 7.4, fixed with buffered 4% paraformaldehyde for 16 h at 4 °C, and rinsed twice with 0.1 M phosphate buffer. Then, they were embedded in 5% agar (Super LM; Roth), in 0.1 M phosphate buffer. Then, 50 μm floating sections were cut with a vibratome (Thermo Scientific Microm HM 650 V) and mounted on microscope slides. They were incubated for 5 min in PBS/0.5% Triton X-100 (Sigma Aldrich), rinsed in PBS, incubated for 30 min at 37 °C in PBS/0.2% hyaluronidase (Sigma Aldrich), rinsed in PBS, and treated with PBS/10% bovine serum albumin (BSA; Sigma Aldrich). Immunohistochemical staining was carried out using polyclonal-specific antibodies against type I and type II collagens diluted to 1:100 (Novotec). These primary antibodies were revealed using a goat anti-rabbit IgG conjugated to Alexa Fluor 546 diluted to 1:2000 (Invitrogen). As controls, primary antibodies were omitted. Slides were then treated with UltraCruz Mounting Medium (4,6-diamidino-2-phenylindole, dilactate; Santa Cruz Biotechnology, Inc.). Observations were made on a confocal laser-scanning microscope (Olympus FV1000). The same parameters were used for all acquisitions, and representative pictures are shown in the figures.

### Immunohistochemistry

Constructs were fixed with 4% paraformaldehyde for 16 h, dehydrated using successive baths of graded ethanol, embedded in paraffin, and sectioned in 4 μm-thick slices. Immunostaining was performed as previously described^27^. Briefly, sections were deparaffinized using xylene and rehydrated in ethanol. Immunostaining was initiated by unmasking the antigenic sites with 0.5% hyaluronidase in PBS-BSA buffer (3%), washed with PBS and permeabilized. Then, the sections were treated with polyclonal-specific antibodies against type I (1:1 000 dilution) and type II (1:250 dilution) collagens and aggrecan (1:500 dilution). Following washings with PBS, sections were then treated with 1.5% hydrogen peroxidase in PBS-BSA followed by HRP-conjugated secondary antibody (undiluted, EnVision+; Dako), and signals were detected using the DAB substrate (Dako). Hematoxylin (Labonord SAS) was used as the counter-stain. An Aperio ScanScope slide scanner was used to digitalize histological slides (Leica Biosystems). Alternatively, sections were also stained with hematoxylin-eosin-safran (HES), alizarin red S (2%, pH 4.1; Sigma) and Alcian blue (1% pH 2.6; Sigma) according to routine protocols.

### Statistical analysis

All experiments were repeated at least four times with cells from different donors. Values are reported as means ± SD or box plots. Statistical analyses were performed using the Mann-Whitney U-test to determine significant differences between two groups. For multiple comparisons, groups were compared using the Kruskall-Wallis test. All statistical analyses were done using Prism v5.0a (Graphpad, San Diego, CA, USA). A P-value of ≤0.05 was considered to be significant.

## Additional Information

**How to cite this article**: Gómez-Leduc, T. *et al*. Chondrogenic commitment of human umbilical cord blood-derived mesenchymal stem cells in collagen matrices for cartilage engineering. *Sci. Rep*. **6**, 32786; doi: 10.1038/srep32786 (2016).

## Supplementary Material

Supplementary Information

## Figures and Tables

**Table 1:** Comparison of the expression of surface markers of UCB-MSCs from passage two to eight.

	CD29	CD44	CD73	CD90	CD105	CD146	CD166
P2	98.4 ± 2.03	100 ± 0.07	98.8 ± 1.44	72.7 ± 27.8	93.4 ± 2.37	41.1 ± 37.8	98.7 ± 0.75
P3	99.3 ± 0.62	99.9 ± 0.34	99.3 ± 0.66	81.5 ± 30.1	92 ± 9.36	69.5 ± 21.3	97.5 ± 3.35
P4	97.3 ± 5.23	99.9 ± 0.19	99.2 ± 1.07	77 ± 24.3	91.9 ± 10	50.5 ± 29.5	97.4 ± 1.8
P5	98.6 ± 2.04	99.9 ± 0.09	97.9 ± 3.14	60.2 ± 32.6	92.1 ± 9.76	51.3 ± 26.6	93.9 ± 5.54
P6	98.4 ± 1.70	99.8 ± 0.35	99.2 ± 1.19	81.4 ± 36.1	87.8 ± 11.0	24.1 ± 23.3	98.5 ± 0.94
P7	96.4 ± 3.44	99.5 ± 0.62	96.6 ± 3.53	93.4 ± 8.69	91.5 ± 4.24	25.2 ± 22.8	97.4 ± 2.28
P8	98.0 ± 0.93	100 ± 0.05	99.4 ± 0.85	79.5 ± 39.1	80.5 ± 25.2	26.5 ± 33.5	98.2 ± 0.50

The table shows mean values of the percentage of positive cells ± standard deviation for the total number of cells analyzed (n = 7).

**Table 2:** Primers used for RT-qPCR.

Genes	Primer sequence (5′-3′) (F: Foward; R: Reverse)
*SOX9*	F: CCCATGTGGAAGGCAGATG
	R: TTCTGAGAGGCACAGGTGACA
*COL2A1*	F: GGCAATAGCAGGTTCACGTACA
	R: CGATAACAGTCTTGCCCCACTT
*ACAN*	F: TCGAGGACAGCGAGGCC
	R: TCGAGGGTGTAGCGTGTAGAGA
*COL2A*	F: TGCAGGATGGGCAGAGGTATA
	R: GAGGCAGTCTTTCACGTCTTCAC
*COL2B*	F: CCGCGGTGAGCCATGA
	R: TTTGGGTCCTACAATATCCTTGATG
	TaqMan probe: FAM-CCA GGA TGT CCG GCA ACC AGG A-TAMRA
*COL1A1*	F: CACCAATCACCTGCGTACAGAA
	R: CAGATCACGTCATCGCACAAC
*COL10A1*	F: AAACCAGGAGAGAGAGGACCATATG
	R: CAGCCGGTCCAGGGATTC
*MMP13*	F: AAGGAGCATGGCGACTTCT
	R: TGGCCCAGGAGGAAAAGC
*RUNX2*	F: GTACAGCTTTAAGGATTCCCTCAATTC
	R: TTGCTAATGCTTCGTGTTTCCA
*OSTEOCALCIN*	F: CGGTGCAGAGTCCAGCAAA
	R: GGTAGCGCCTGGGTCTCTTC
*RLP13a*	F: GAGGTATGCTGCCCCACAAA
	R: GTGGGATGCCGTCAAACA
