# Supplementary material for: Chondrogenic commitment of human umbilical cord blood-derived mesenchymal stem cells in collagen matrices for cartilage engineering
Source: Sci Rep. 2016 Sep 8;6:32786. doi: 10.1038/srep32786 (PMC5015060; doi:10.1038/srep32786)
Supplement: Supplementary Information [file srep32786-s1.pdf]

Table S1: Characteristics of the 8 (1-8) samples of hUCB-MSCs used in this study among 15. Empty boxes = not determined.

Chondrogenic commitment of human umbilical cord blood-derived mesenchymal stem cells in collagen matrices for cartilage engineering

Tangni Gómez-Leduc, PhD,[1] Magalie Hervieu, PhD,[1] Florence Legendre, PhD,[1] Mouloud Bouyoucef, PhD,[1] Nicolas Gruchy, MD PhD,[1,2] Laurent Poulain, PhD,[3] Claire de Vienne, MD,[4] Michel Herlicoviez, MD PhD,[4] Magali Demoor, PhD,[1,†] Philippe Galéra, PhD, [1,†,*]

				MSCs markers							Hematopoietic markers					Differentiation capacity		
	**Sample volume (ml)**	**Day for first colony**	**Population doubling P3**	**CD29**	**CD44**	**CD73**	**CD90**	**CD105**	**CD146**	**CD166**	**CD14**	**CD34**	**CD45**	**CD64**	**HLA-DR**	**Osteoblast**	**Adipocytes**	**Chondrocytes**
**1**	103	10	3	99.5	100	98.8	94.1	99.1	87.1	99.2	<1	<1	<1	<1	<1	+	+	+
**2**	40	13	2.89	99.5	100	98.4	99.9	90.1	64.7	98.4	<1	<1	<1	<1	<1	+	-	+
**3**	60	13	2.32	99.6	100	99.9	67.1	94.9	18.2	94.4	<1	<1	<1	<1	<1			+
**4**	61	14	2.49	99.9	100	100	85.6		41.6		<1	<1	<1	<1	<1			+
**5**	33	12	2.16	99.6	100		70.9				<1	<1	<1	<1	<1			+
**6**	63	14	2.63	99	99.2	99.3	99.6	93.1	15.5	97.5	<1	<1	<1	<1	<1	+	-	+
**7**	59	9	2.21	98.1	99.1	99.5	99.5	76.6	38.6	99.1	<1	<1	<1	<1	<1	+	+	+
**8**	50	13	2.46	99.7	100	100	100	99	91.9	100	<1	<1	<1	<1	<1	+	+	+
**9**	65	14	2.60	99	100	97.2	28.1	92.4	74.9	97.9	<1	<1	<1	<1	<1			
**10**	68	14	3.13	93.5	100	99.9	81.7				<1	<1	<1	<1	<1			
**11**	53	15		98.9	100	100	65.6				<1	<1	<1	<1	<1			
**12**	158	5	2.87	99	100	99.9	100	91.4	5.2	98.8	<1	<1	<1	<1	<1			
**13**	26	23	2.92	100	100		50.5				<1	<1	<1	<1	<1			
**14**	33	11		96.6	100		99.5				<1	<1	<1	<1	<1			
**15**	48	23	2.58	95.5	99.5		67.4				<1	<1	<1	<1	<1			
